# Realization of a Family Systems Care Unit: A Real-Life Laboratory for Clinical Practice, Education, and Research at a Swiss University of Applied Sciences

**DOI:** 10.1177/10748407251411202

**Published:** 2026-01-16

**Authors:** Evelyn Huber, Margrit Hilpertshauser, Corina Sgier, Elisabeth Stark, Katharina Fierz, Sonja Bächi, André Fringer, Barbara Preusse-Bleuler

**Affiliations:** 1 ZHAW Zurich University of Applied Sciences, Winterthur, Switzerland

**Keywords:** Family Systems Care, family nursing, real-life laboratory, project management, action learning

## Abstract

The Family Systems Care Unit (FSCU) is a real-life laboratory including a counseling service for family-systems-centered therapeutic conversations with families with burdening health issues. Health care students and professionals observe these conversations for vicarious learning. Video-recorded conversations are used for research and educational purposes. Since 2020, the FSCU has been developed using project management and action learning strategies. As of June 2025, 34 families had used therapeutic conversations. Fifty health care students and professionals observed conversations and participated in the clinical team’s pre- and post-sessions. Thirteen students completed their master’s theses. A case vignette illustrates a family confronted with one family member’s decision to obtain medical aid in dying, demonstrating the interconnectedness of the FSCU’s clinical work, education, and research. The FSCU is a care model that addresses today’s health care and higher education needs. The objectives of this article are to present how this FSCU was realized over a 5-year project period.

## Introduction

Twenty-five years ago, the World Health Organization (WHO, 2000) introduced the “Family Health Nurse” role. The primary objective of this role was to provide care to individuals and families experiencing illness, disability, or other health-related challenges within the context of their homes and communities, as well as during transition periods. This care should consider the various systems, interactions, and complexities implicated. In addition, the WHO (2015, 2016) emphasized the need for more people-centered and integrated care to address contemporary, complex health challenges. People-centered care integrates the views, needs, and preferences of individuals, families, and communities. Integrated care refers to a health care system that delivers comprehensive services to individuals and their families throughout their lives, addressing their diverse needs and conditions (WHO, 2015, 2016). Family members define who belongs to “the family,” regardless of whether they are genetically or legally related or not (Hriberšek et al., 2024; Rolland, 2025; Shajani & Snell, 2023).

Since 2000, Switzerland has trained advanced practice nurses (APNs) in accordance with the WHO (2005), which recommends expanding the skill sets of health care professionals (Schwendimann et al., 2019). APNs are educated to care for patients and their families across the care spectrum and respond to complex health care needs (Schober et al., 2020). Moreover, the International Family Nursing Association (IFNA, 2017) introduced “Family Nursing-Advanced Practice,” defined as a “focused application of an expanded range of nursing competencies to improve health outcomes for patients and families in the larger discipline of nursing” (p. 2).

However, cultivating expanded competencies requires modifications to higher education in health care (Fringer, 2021; Jonnergård et al., 2024; Schwendimann et al., 2019). APNs require a multifaceted set of clinical and research skills to provide comprehensive care to patients and their families and investigate the benefits of clinical interventions in all their complexity, particularly within the community context (Fringer, 2021). Competencies in interprofessional collaboration (Mohammed et al., 2021), people-centered care, communication and interpersonal skills, and professional aspects such as commitment, resilience, awareness, and reflection must be learned (Ryan, 2022). Active learning situations that provide opportunities to observe, model, and reflect on clinical practice within safe yet challenging learning environments facilitate the development of these competencies, skills, and qualities (Stuart et al., 2021). Consequently, lecturers are encouraged to adopt a role-model approach, and students are expected to assume the roles of transformers and multipliers when they enter clinical practice (Jonnergård et al., 2024).

To contribute to the implementation of this paradigm shift, the Institute of Nursing (IN) at a Swiss University of Applied Sciences’ School of Health Sciences (SHS) established an APN-led interprofessional Family Systems Care Unit (FSCU) as a real-life laboratory (lab). A real-life lab is a space dedicated to user-centered and co-creative knowledge sharing, innovation development and testing, and research (Archibald et al., 2021; Zipfel et al., 2022). The FSCU is a real-life lab that integrates clinical counseling for families with real-life illness-related burdens with family systems care (FSC) education, training, and research (Huber et al., 2024; Preusse-Bleuler & Stark, 2021).

The clinical counseling method used at the FSCU is referred to as “therapeutic conversations” (Shajani & Snell, 2023; Wright & Bell, 2021). These conversations are family-systems-centered, collaborative, and strength-oriented and aim to provide support for the management of health and illness issues in everyday life, alleviate illness-related suffering, and promote the health and healing of individual family members and families as a whole (Bell, 2008; IFNA, 2017; Preusse-Bleuler, 2025; Wright & Bell, 2021). Such therapeutic conversations have been shown to yield several benefits for the families. These benefits include increased awareness of the family’s strengths and needs, an enhanced ability to cope with the patient’s situation (Mileski et al., 2022), and improved family functioning (Azcárate-Cenoz et al., 2024; Mileski et al., 2022). In addition, families have reported increased professional support and improved quality of life (Azcárate-Cenoz et al., 2024).

The FSCU is based on the pioneering Canadian Family Nursing Unit at the University of Calgary. Dr. Lorraine M. Wright spearheaded the inception of this unit in 1982, and she and Dr. Janice M. Bell oversaw it for 25 years (Bell, 2008). The Family Nursing Unit in Calgary is widely regarded as the progenitor of FSC in nursing, due to its significant contributions to developing and advancing family nursing models based on empirical findings and scientific inquiries into nurse-led family-systems-centered therapeutic conversations (Bell, 2008). These models include the “Calgary Family Assessment and Intervention Models” (Shajani & Snell, 2023), the “Illness Beliefs Model” (Wright & Bell, 2021), and the “Trinity Model” (Wright, 2017). These models facilitate the identification, acknowledgment, and response to the multifaceted interconnections between health conditions, ill individuals, involved family members or close persons, community structures, and societal circumstances in therapeutic conversations (Bell, 2008).

The concept of the faculty-based Family Nursing Unit at the University of Calgary (Bell, 2008) was adopted by a few universities, including the Center of Excellence in Family Nursing at the University of Montreal in Canada (Duhamel et al., 2015) and the Family Care Unit at Kalmar University in Sweden (Benzein et al., 2008). To the best of our knowledge, there have been no recent reports on the implementation of family nursing units. Therefore, this article presents the 5-year project period for conceptualizing and developing the Swiss FSCU and its achievements over the past years. This information may benefit health care professionals engaged in analogous strategic initiatives or service development.

## Project Description

### Project Aims and Objectives

According to Preusse-Bleuler and Stark (2020), the FSCU’s overall aim has been to develop answers for today’s health and societal challenges related to promoting the health, well-being, and quality of life of families affected by health and illness issues, as well as family caregivers at risk of becoming ill themselves. The aims of the 5-year project period were to establish the FSCU as a real-life lab and competence center for FSC clinical practice, education, and research with the following objectives:

- To promote sustainable interprofessional FSC expertise and clinical competencies.
- To develop a setting for innovative teaching methodologies and advance educators’ competencies.
- To create a counseling service for individuals and families dealing with health and illness issues and for professionals seeking FSC knowledge and skills.
- To provide clinical data on expert practice for FSC research and educational research.

### Context and Setting

The SHS, to which the FSCU belongs, was founded in 2006. Since its inception, its IN has incorporated family nursing modules into its curriculum, drawing upon the Calgary Family Assessment and Intervention Models (Shajani & Snell, 2023), the Illness Beliefs Model (Wright & Bell, 2021), and the Trinity Model (Wright, 2017). These modules have been conceptualized and taught by the last author (BPB) at the Bachelor of Science (BSc) and Master of Science (MSc) levels and in continuing nursing education programs since 2007. Later, FSC was taught jointly to midwives and nurses at the MSc level. Integrating high-fidelity simulation training that incorporates FSC further enhances the student’s self-efficacy (Häusermann et al., 2023, 2025). The IN boasts two professorships in family-centered care and one in community care. At 2-year intervals, the IN invites Dr. Lorraine M. Wright and Dr. Janice M. Bell to direct a summer school program that is open to health care professionals and students from within and outside the SHS.

Following a visit to the Family Nursing Unit in Calgary (Bell, 2008) in the early 2000s, BPB aspired to advance the FSC education and research initiatives by establishing a comparable unit in Switzerland. Upon the SHS’s relocation to the present building in 2020, BPB began a project to conceptualize and establish the FSCU within the novel and distinctive faculty practice unit. This unit comprises two rooms interconnected by a one-way window, a video transmission system, and video-recording technology. One member of the clinical team offers therapeutic conversations to families. Assuming informed consent, we record the therapeutic conversations on video for education and research. Groups of additional clinical team members, nursing students, SHS lecturers and researchers, and health care professionals from external partner institutions observe the conversations from the adjacent room through the one-way window. Each therapeutic conversation is prepared for and evaluated by the clinical team members and guests present. The guests are invited to observe conversations for vicarious learning, mutual reflection, quality assurance, and research. The pre- and post-sessions are also video-recorded.

### Project Design

We integrated project management strategies with a participatory action learning framework to realize this project, as the FSCU team was formed to serve as the project team.

A project has been defined as a “temporary endeavour undertaken to create a unique product, service, or result,” and its deliverables may endure beyond the project period (A guide to the project management of knowledge [PMBOK guide] and the standard for project management, 2021, p. 25). Project management involves applying knowledge, skills, tools, and techniques to achieve a project’s goals. Successful project management necessitates diligent, respectful, and caring leadership that facilitates collaborative and resilient teamwork. It also requires stakeholder engagement, building on common values and a shared vision. In addition, the context, the quality of the deliverables, and the project’s complexity and risks need to be considered.

Action learning is a collaborative approach in which a group works together to learn and solve problems (Hurst & Marquardt, 2019). It consists of iterative elements: questioning assumptions, acting, reflecting on the action, and asking new questions (Hurst & Marquardt, 2019). According to Zuber-Skerritt and Wood (2019), action learning is based on a participatory, systemic, and holistic paradigm. This paradigm recognizes that knowledge is created within social groups and seeks to understand reality by sharing experiences. Action learning aims to understand perspectives, address challenging circumstances, and advocate for the individuals who benefit from the action learning process. Project team members and facilitators collaborate to solve shared concerns, acknowledging the facilitators’ inherent subjectivity as an integral component of the process. Action learning is relational, transformational, and evolutionary, thus aligning with the concept of a real-life lab.

### Project Application

In 2019, BPB initiated preparatory work by engaging with stakeholders, including the IN head, the heads of IN departments, the SHS head, and other lecturers and researchers using the SHS faculty practice unit. Based on their commitment and the university’s approved special funds, BPB became the co-head of the FSCU and was responsible for the project’s professional and scientific content. ES was assigned as the administrative co-head. Consequently, they collaborated in formulating a project application for the IN head’s attention. This application encompassed the FSCU’s vision and objectives, the SHS’s fundamental conditions, an analysis of internal strengths and weaknesses and external opportunities and threats (SWOT; Berthold, 2009), a 5-year project plan, an organizational chart, a 2-year budget, and a work plan for a standard FSCU workday (Preusse-Bleuler & Stark, 2020). The IN head approved the application in May 2020.

#### Vision

**Figure 1.**
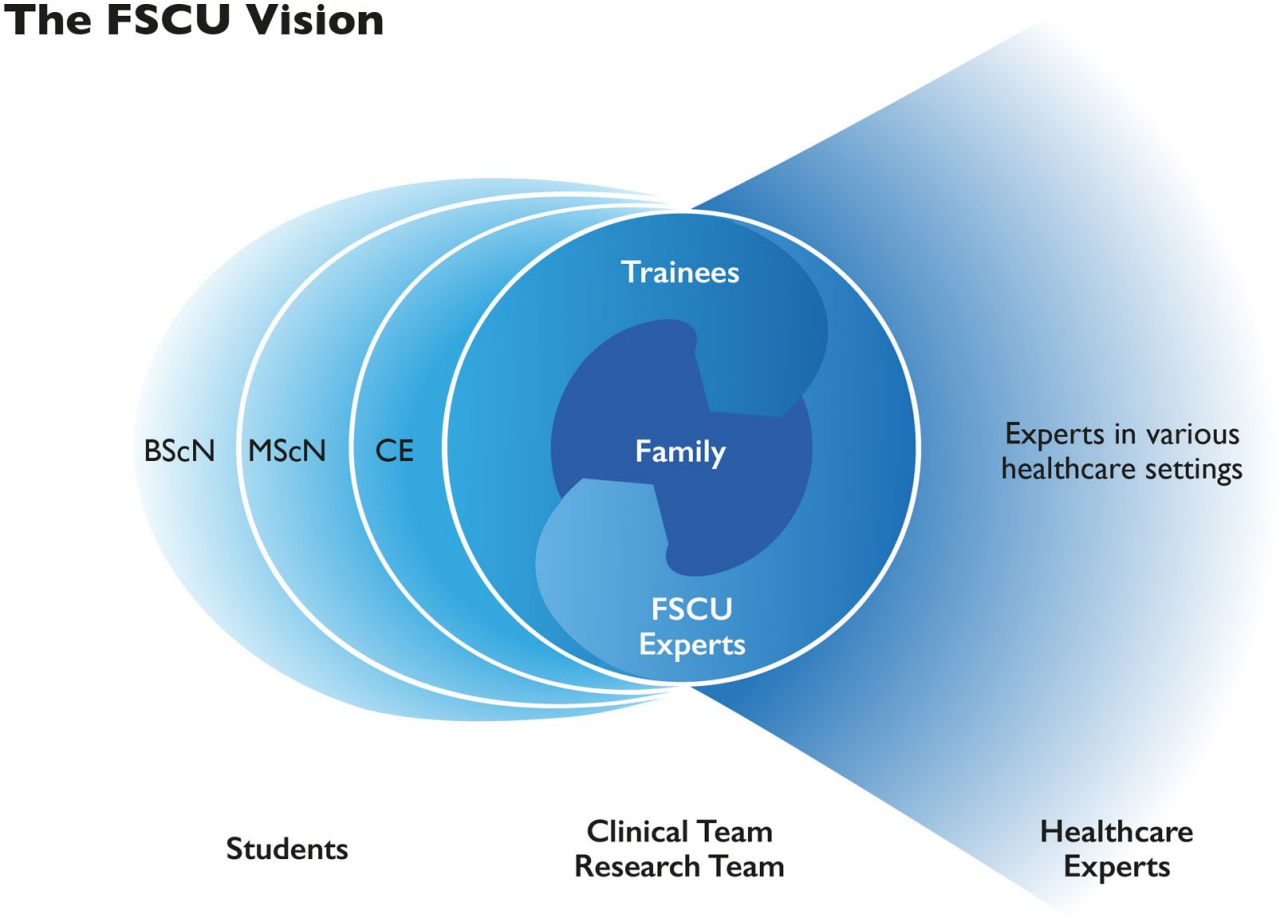
The FSCU Vision. *Note.* This figure was created by Barbara Preusse-Bleuler in 2018. BScN: Bachelor of Nursing Science; MScN: Master of Nursing Science; CE: continuing education.

The FSCU vision is based on the SHS mission, which includes five topics: (a) innovative health care solutions, (b) professional health care expertise for individuals and groups, (c) interprofessional collaboration, (d) applied education and research, and (e) co-creative solutions with those affected from health care problems (Guiding Star School of Health Sciences, 2024; Preusse-Bleuler & Stark, 2020). BPB translated this mission into the FSCU vision, as illustrated in Figure 1.

**Figure 2.**
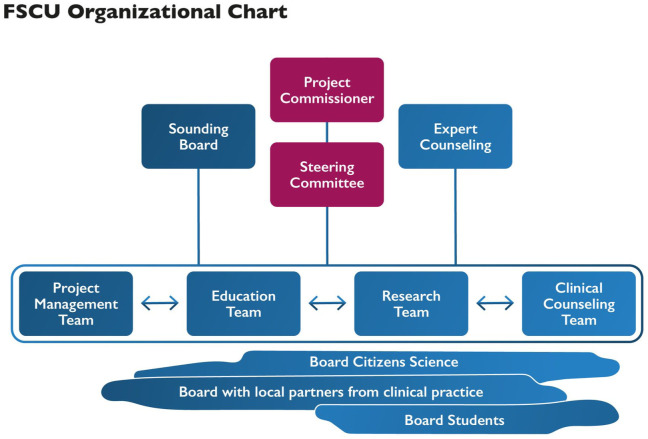
FSCU Organizational Chart.

1. As an innovative FSC real-life lab, the FSCU occupies the central position in the figure. Clinical family health care experts offer therapeutic conversations to families with real-life illness-related burdens while training future experts in a post-master’s trainee program. In this program, trainees observe and conduct therapeutic conversations under the supervision of experts and peers to become multipliers of FSC in various health care settings represented by the funnel on the right side of the figure.
2. The FSCU is an APN-led initiative that applies and advances FSC skills and knowledge based on the established family nursing models as described by Shajani and Snell (2023), Wright and Bell (2021), and Wright (2017).
3. The clinical and research FSCU teams are interprofessional, benefiting from broader expertise and learning from each other.
4. Applied education and research are supported by learning from real-life family issues and the practical application of counseling methods and skills. As illustrated by the sickles on the left side of the figure, the FSCU’s applied practice is to be included in the BSc and MSc nursing curricula and in continuing nursing and interprofessional education. Video recordings of therapeutic conversations can be used for classroom discussion. Students can observe therapeutic conversations in real time through the one-way window for vicarious learning or analyze video recordings in their MSc theses. Researchers and clinicians investigate and advance FSC knowledge.
5. Co-creation is realized through non-hierarchical, participatory conversation methods that support families in finding solutions that suit their situation. These methods are also used in co-creative pre- and post-sessions of therapeutic conversations with groups of clinical experts, trainees, researchers, students, and professionals who reflect on the preparation for and learning from the conversations.

#### Analysis of Strengths, Weaknesses, Opportunities, and Threats (SWOT Analysis)

The SWOT analysis (Berthold, 2009) revealed the following internal strengths and weaknesses, as well as external opportunities and threats regarding the project realization:

- Strengths: FSC expertise in clinical practice, practice translation, research, and higher professional education; experience in acquisition; and dedication to fostering collaboration between research, education, and practice.
- Weaknesses: Lack of prior experience with such a unit; limited implementation of interprofessional cooperation; a limited number of young experts and key knowledge holders; complexity due to numerous interfaces; and financial constraints of the project.
- Opportunities: Pioneering initiative with the potential to act as a strategic magnet for the university; developing an FSC counseling profile as a distinct nursing competency; and advancing interprofessional FSC as a catalyst for strengthening community care and accelerating further health care innovations.
- Threats: Legal uncertainties regarding the feasibility of combined teaching and service; high complexity due to the coordination of the multiple interfaces; lack of donor outreach thus far; and competition with local service providers (Preusse-Bleuler & Stark, 2020).

The SWOT analysis identified strategic challenges requiring expansion, safeguarding, mitigation, or avoidance, informing the derivation of goals and measures for a 5-year project plan.

#### Project Plan

The project plan spanned from the summer of 2019 to the summer of 2025 (Preusse-Bleuler & Stark, 2020). The 2019/2020 school year was considered project Year 0, marking the preparatory work necessary to begin operating the FSCU. School years 2020/2021 to 2024/2025 detailed the work packages for developing the five domains: (a) overall organization, (b) family counseling, (c) education, (d) research, and (e) dissemination. Stage one covered developing the operational FSCU structure during the project Years 0 through 3. Stage two included the consolidation, evaluation, expansion, and network promotion during project Years 4 and 5.

#### Organizational Chart

Figure 2 shows the FSCU organizational chart, which illustrates the areas of responsibility and the hierarchy of collaboration: sounding board, project commissioner, steering committee, project teams, boards, and expert counseling. The FSCU team comprises the two FSCU co-heads, who are responsible for project management and facilitating action learning processes; the FSCU clinical experts, who provide therapeutic conversations and translate FSCU practices into SHS education; and the research team. Experts from the former Family Nursing Unit in Calgary (Bell, 2008) comprise the expert consultation board. Boards consisting of citizens, practice partners, and students should support the development and operation of the FSCU.

### Ethical Considerations and Data Protection

The Kanton Zurich Ethics Committee confirmed that this project realization and the clinical FSCU work do not fall under Swiss human research legislation (waiver no. Req-2021-01424, December 14, 2021). However, we have carefully followed good clinical practice guidelines and Swiss data protection laws, given the highly sensitive real-life family data. First and foremost, all family members and the individuals present during pre- and post-sessions or other meetings that are recorded on video are asked to sign an informed consent form offering different levels of agreement regarding the handling of personal data and video recordings, the use of video recordings for educational purposes, the use of video recordings and other data for research purposes, and the use of analyzed and anonymized data for dissemination purposes. We recently updated our informed consent forms to detail the processes for adult, adolescent, and minor family members, as well as for students and professionals. Our university’s internal ethics commission reviewed these forms and approved them for a period of 3 years (EA-ZHAW 2024-027-G).

All students and professionals with access to the families’ data sign a form regarding the obligation for professional secrecy. We provide all families with a numeric code and an alias for internal communication, and only the two FSCU co-heads and one clinical expert have access to the key file. All electronic data is stored in secure, restricted folders on our university’s server. We store paper documents in a locked cabinet inside the SHS faculty practice. More information is provided in Huber et al. (2024).

## Project Outcomes

### Status of the Project Plan Realization

In the following, we describe what we have achieved during the 5-year project period, along with the five project domains defined in the project plan.

#### Project Domain 1: Overall Organization

Clinical team members interested in establishing the FSCU project through an action learning collaboration process were recruited from among the lecturers and researchers of the IN’s BSc and MSc programs. Initially, the team included one APN, one nurse and sociologist, and a family physician from the IN, as well as one external APN. After 2 years, one clinical expert retired and was replaced by another APN. All of them have received further training in areas such as systemic counseling. They all work part-time for the FSCU, which initially equalled one full-time position, extended to one and a half full-time positions by the fifth year, plus extra hours for subprojects.

**Figure 3.**
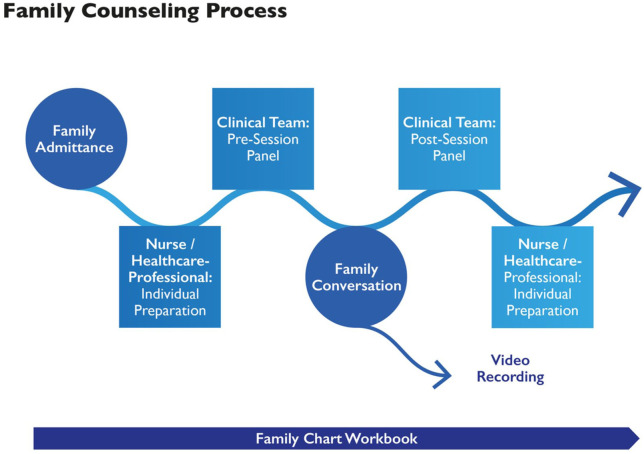
Family Counseling Process. *Note.* This figure was created by Barbara Preusse-Bleuler in 2018.

Using the action learning approach, the co-heads and the clinical team members have continuously developed and advanced organizational processes and content as needed. Initially, they met every one to 2 weeks, and later every 4 to 6 weeks. The team also worked in-depth during annual 2.5-day retreats and received external expert consultation from Dr. Lorraine M. Wright and Dr. Janice M. Bell once a year. Examples of products that were created and refined include:

- The workflow and content of the therapeutic conversation units (see Figure 3).
- The process of working clinically in tandems for quality assurance and mutual learning.
- The clinical workbook documenting the counseling processes, including a genogram-ecomap (Shajani & Snell, 2023); important information such as family members’ medical diagnoses; summaries of the therapeutic conversations; and hypotheses on the family’s strengths, needs, health and illness beliefs, and red flags, as reflected in the pre- and post-sessions.
- The use and handling of the technical equipment of the FSCU premises (one-way window, video recording, and sound transmission system).
- The handling of ethical standards and data protection laws, as described above.
- The creation of informational materials, such as a webpage and a brochure.
- The elaboration of a project evaluation plan.
- Guidelines for using family conversations in lectures, publications, and research.

In addition, the co-heads and the team prepared oral presentations and biannual status reports for the IN head. They revised the SWOT analysis after 2 years and at the end of the fifth year. Furthermore, after 3 years, the FSCU was incorporated into the IN’s organizational structure as its unit and assigned to the IN’s MSc and research department.

#### Project Domain 2: Family Counseling

Figure 3 shows the process of therapeutic conversations utilizing a systemic approach. We tested this process with two families at the FSCU premises and two in their homes in 2020. We started regular therapeutic conversations in 2021, first online due to the COVID-19 pandemic and later at the SHS premises. If required, we also conducted conversations in families’ homes, hospitals, or palliative care hospices.

Families typically find the FSCU through the internet or personal contacts. For example, a former student, a health care professional, or a health insurance provider may refer them to the FSCU (Huber et al., 2024). In exceptional cases, we have recruited families from specific populations by contacting health or social organizations for research purposes. To date, the number of families seeking to use the FSCU service exceeds our capacity due to limited financial and staff resources, as well as shared premises with other SHS staff.

When a family contacts the FSCU, a clinical team member calls to determine whether the FSCU service can address their concerns before scheduling the first appointment. We organize therapeutic conversations according to the families’ concerns and preferences to be discussed, following the Calgary Family Assessment and Intervention Model (Shajani & Snell, 2023), the Illness Beliefs Model (Wright & Bell, 2021), and the Trinity Model (Wright, 2017). At the end of each conversation, the clinical expert asks the family members present how the conversation went, if they would like to have another conversation, and what they would like to discuss next. Some therapeutic conversations are supplemented by writing a therapeutic letter to the family. These letters acknowledge the family’s or individual’s suffering and strengths, as well as notable issues from the therapeutic conversations, and express commendations (Wright & Bell, 2021).

Therapeutic conversations are free for families because we ask that they be recorded for educational and research purposes. Currently, APN counseling services are not covered by mandatory basic health insurance or voluntary supplementary health insurance in Switzerland.

#### Project Domain 3: Education

The FSCU team developed and implemented educational activities for nursing and midwifery students, SHS professionals, and external practice partners, including hospital APNs and health insurance case managers. These activities include observing therapeutic conversations through the one-way window and actively participating in the pre- and post-sessions. For instance, we invite MSc nursing students to observe therapeutic conversations during an internship at the FSCU or to write their MSc thesis by analyzing video recordings of these conversations.

At the BSc and MSc levels, as well as in continuing education, clinical team members who are also lecturers have developed teaching units that show and discuss selected video recordings in the classroom. In addition, BPB has initiated an interprofessional continuing education course on the basics and advanced fundamentals of FSC, in collaboration with interprofessional colleagues, utilizing FSCU material. Furthermore, we designed and pilot-tested post-master training for trainees to moderate therapeutic conversations with families under the supervision of clinical team members.

#### Project Domain 4: Research

We formed a research team of two nursing scientists, one health scientist, and one sociologist. All of them are trained as nurses and work as SHS lecturers and researchers. Every 2 to 3 months, they meet with the two FSCU co-heads and clinical team members in a research group meeting to discuss the acquisition of third-party funds and research and dissemination activities.

As outlined in more detail by Huber et al. (2024), the research activities mainly include single and multiple case studies to investigate in depth the strengths, challenges, and needs of families who take advantage of therapeutic conversations, how these conversations support these families, and the mechanisms of the conversation techniques used. Such research activities have been conducted since the beginning of the FSCU project. Case study research helps us to learn from the complexity of individual situations and understand the impact of APN care in such situations (Fringer, 2021; Wildhaber et al., 2025). In the context of a 5-year FSCU project evaluation, we are investigating how the FSCU setting supports student and professional learning through focus group interviews, as well as how families describe the effects of family conversations at the FSCU using family interviews. Researchers, clinicians, and students collaborate to enhance reflexibility and research rigor (Rettke et al., 2018).

Simultaneously, we have been writing various applications for third-party funding. Due to a lack of third-party funding thus far, we are waiting to begin more comprehensive research projects.

#### Project Domain 5: Dissemination

Regarding dissemination activities, members of the FSCU clinical and research team have presented the FSCU, its methods, and preliminary research findings at various national and international conferences, including the IFNA Conference in Dublin in 2023 (Horowitz & Neill, 2024). We have also published articles in peer-reviewed journals (Huber et al., 2024; Sgier et al., 2025; Wildhaber et al., 2025), non-peer-reviewed journals (Edmaier & Preusse-Bleuler, 2024; Hilpertshauser et al., 2024; Preusse-Bleuler, 2025), and have provided workshops and informational events at the SHS for colleagues from the SHS and external partner institutions. Furthermore, we have established networking activities within the SHS and external organizations. We wrote one non-peer-reviewed journal article in collaboration with a family member after using therapeutic conversations at the FSCU (Hilpertshauser et al., 2024).

### Outputs Reached

#### Family Counseling

As of June 2025, 57 families had contacted the FSCU. Of those families, 34 used family-systemic therapeutic conversations, and 15 received triage meetings to determine if the FSCU service would meet their needs. Eight families who contacted the FSCU did not have therapeutic conversations because the problems were resolved or the requests required the attention of professionals outside the FSCU clinical team. Of these 57 families, 29 were parents of minor children, and eight had a family member above 65. Nine families came with four to six members, 15 came with three members, and eight came with two family members. Eight individuals came alone. The age range of those for whom the family was primarily seeking therapeutic conversations was 3 weeks to 92 years old.

We provided a total of 177 therapeutic conversations. Of the 34 families who used therapeutic conversations, the median number of therapeutic conversations was three, and the mean was 5.2 due to one family needing long-term support. Sixty-five therapeutic conversations took place at the FSCU premises, and four at another university location. Twenty-three conversations took place at the families’ homes, and 43 online due to the COVID-19 pandemic or because the families could not travel because of illness, having small children, or family members living far apart. Forty-two family conversations were conducted using video conferencing, phone calls, or written communications via email.

The families’ health and illness-related concerns were discussed in the context of the following relational constellations: couple relationships (19), parent–child relationships with minor children (16), family as an entity (9), parent–child relationships with adult children (4), family members’ concerns in the context of a challenging family situation or duty (3), friends (2), no details provided (2), patient–physician relationships (1), and one family caregiver’s stressful relationship with a team of health care providers (1).

The families’ concerns brought to the therapeutic conversations involved a large variety of challenges and needs. Some families sought conversations to gain a better understanding of their situation, needs, or options, to be accompanied through a process or transition, or to discuss issues and find new solutions within the family (27). Others looked for information (8) or practical support (5). The families expected the conversations to bring more clarity in their understanding of the family’s or a family member’s situation and options for action by finding support in health-related transitions (17), renegotiation of relationships (10), struggles with domestic violence (2), or conflicts with health care professionals (2). Finally, some families volunteered for the FSCU by contributing their perspectives and experiences for students’ MSc theses or research interests (6).

Families with psychological distress or psychiatric symptoms make up a large proportion of the families who received therapeutic conversations at the FSCU. This indicates an unmet need for support in such families.

#### Education and Research

Fifty students and professionals observed therapeutic conversations through the one-way window and participated in pre- and post-sessions. Six classes worked with video-recorded therapeutic conversations. Two students completed internships in education and five in research. One APN and one advanced practice midwife pilot-tested the FSCU post-master training program.

Thirteen students have completed their MSc theses and are preparing them for publication. One MSc thesis was recently published (Wildhaber et al., 2025). Seven students are currently working on their theses. Examples of the topics include:

- The effect of commending as an FSC intervention on a family and the process of therapeutic conversations.
- The challenges and needs of families raising a child with a disability and how they benefited from a series of therapeutic conversations (Wildhaber et al., 2025).
- Facilitation of family resilience in a family experiencing the departure of adult children from the parental home in the context of the mother’s and one daughter’s serious illness.
- Developing a practical model of family resilience based on three families’ experiences: one with stillbirth, one with the early death of a parent, and one with medical aid in dying (MAiD).
- The experience of a serious illness as a couple, such as an acute myocardial infarction or multiple sclerosis.
- Core beliefs held by families and FSCU clinical experts and their interrelation with resilience and experiences of suffering.

Furthermore, the FAMETHI (FAMily Systems Care and ETHIcs) research project investigated the best ways to support families affected by early parental loss as part of a doctoral thesis (Sgier et al., 2025). The project aimed to develop expert consensus among the clinical FSCU experts and further health care professionals from the SHS and other health care organizations. The project’s objective was to identify the ethical dimensions inherent to professional FSC practice with attention to health care professionals’ attitudes and beliefs regarding their interactions with families.

### Merging of Clinical Practice, Education, and Research by the Example of a Case Vignette

In a case vignette, we illustrate how clinical practice, education, and research have been integrated at the FSCU and how the FSCU has thus become a real-life lab during the 5-year project.

#### Introduction of the Family Situation

Mr. Casus (84 years old, name anonymized) had his first conversation with an FSCU clinical expert 1 year after his wife passed away. They were married for nearly 60 years and remained very close throughout their lives. Neither of them could imagine living without their beloved partner despite having close relationships with children, grandchildren, and friends. Mr. Casus’ grief over his wife’s death remained unchanged. He deeply missed her despite his family’s presence and support. Previously, Mr. Casus had an active life, always finding ways to solve problems. He enjoyed reading, learning new techniques, and traveling.

His family encouraged him to see an FSCU expert because he was desperately waiting for a committee’s decision to end his suffering by using MAiD “to follow his wife.” MAiD is legal in Switzerland if assisted by an authorized organization and specific rigorous criteria are met beforehand. Waiting for this decision added considerably to his deep grief and suffering. Mr. Casus’ impatience and the intense suffering resulting from the wait and uncertainty of the MAiD admission procedures caused a severe burden and stress for him and his family. The therapeutic conversations mainly focused on how grueling it was to endure such uncertainty for such a long time, as well as the “absurd situation”, which was how the children named it, who wanted to keep their father while also helping him realize his wish to die and end his suffering.

**Figure 4.**
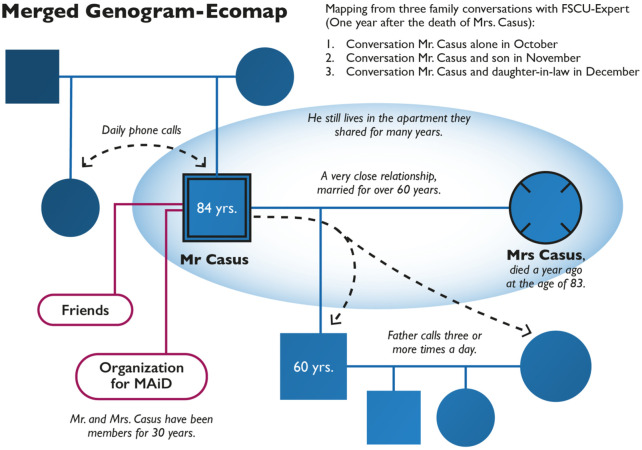
Genogram-Ecomap of Mr. Casus and His Family.

Mr. Casus came alone to the first conversation. He and his son were present at the second conversation. He and his daughter-in-law, who is married to his son, attended the third conversation. His family situation is presented in Figure 4.

#### The Pre-Sessions—Preparation for Conversations Together With Guests

To prepare for the conversations, the FSCU clinical team informed itself on MAiD in Switzerland, for example, by considering the medical-ethical guideline “management of dying and death” by the Swiss Academy of Medical Sciences (2018), and invited guests with experience in various health care settings. Most of them had previously cared for patients who had considered or committed MAiD. Four FSCU experts, two to three students, one IN lecturer, and one external colleague specialized in gerontological community nursing were present during the pre- and post-sessions and observed the conversations. We valued these guests’ questions, concerns, and suggestions, as they provided additional perspectives and helped us prepare for the conversations.

During the first pre-session, we developed the following key hypotheses, which proved helpful in guiding the conversations with Mr. Casus and his family:

- Mr. Casus probably does not want to discuss his decision, but he might be open to discussing how to live until that day.
- Building trust and meaningful conversations will only be possible if Mr. Casus does not feel pressured to justify his decisions or views on life.
- Relatives constantly ask themselves, “Am I doing the right thing?” Ambivalence toward the decision and the decision-making process is a common phenomenon.

#### The Conversations

During the first conversation with the FSCU expert, Mr. Casus described in detail how he and his wife had decided to join a MAiD organization many years ago, how he managed his daily life, and the steps he and his family had taken or still needed to take to utilize MAiD. The waiting and uncertainty of whether he would soon be able to join his deceased wife was difficult for him to bear. At no point did he consider suicide. During the second conversation, which his son also attended, they discussed the family’s ambivalence: they respect their father’s decision but would prefer he take a different path. In the third conversation, Mr. Casus and his daughter-in-law reported on their experiences when she looked for ways to keep her father-in-law alive and how these attempts were ineffective. Mr. Casus appreciated her concerns, and she assured him that she would respect his decision, albeit with a heavy heart. They also discussed the arduous task of repeatedly seeking information about the MAiD organization’s decision-making process and how they, as a family, could help accelerate it. Thus far, the family’s encounters with health care professionals or the MAiD organization have allowed them to discuss only fragments of topics or procedures relevant to the family. At the end of the conversation, they said that having this conversation together brought them relief and helped them better understand their own and each other’s concerns and strategies. The family said the conversations contributed to getting a clearer picture of the whole situation.

#### The Post-Sessions—the Guests’ Perceptions

The conversations touched everyone who observed through the one-way window. During the post-session discussions, the observers’ questions and the FSCU expert’s reflections on the course of the conversation were addressed. Equally important were everyone’s emotional reactions to the topic of MAiD. The students particularly appreciated the opportunity to discuss their questions directly with the experts and gain insight into their considerations regarding the family focus in this situation, as well as multi-person conversations and the course of a conversation about such a sensitive topic. They described their feelings of experiencing the family’s suffering, but also how the clinical team prepared itself for such a conversation and the ethical issues arising from this family’s topic, using words such as “taken” or “deeply touched.”

#### Research

Two MSc theses explored in depth insights from the conversations with Mr. Casus and his relatives: one on family resilience and the other on how “commending” worked as an FSC intervention in these conversations. In addition to advancing their qualitative research skills, the students reported that investigating the video-recorded conversations led to significant personal and professional developments and has become a key element in their development of competence in working with families.

#### Education

Clinical experts, who are also lecturers, developed an educational unit based on selected sequences from these video-recorded conversations. This development was partially carried out in collaboration with a student completing an education internship. We use these counseling sequences in BSc and MSc classes and in workshops with health care professionals. In this educational unit, the health care students or professionals formulate preparatory thoughts and hypotheses with the FSCU expert as they would in a pre-session. Then, they watch the video-recorded conversation sequences together and reflect on them in a post-session. Students experience the FSCU expert as a role model and benefit from their insights into the FSCU expert’s thoughts and considerations before, during, and after the conversation. Furthermore, the FSCU experts and researchers draw on their professional experience with families in their teaching activities, sharing lessons learned in lectures and seminars while consistently maintaining strict anonymity.

In summary, therapeutic conversations are conducted with a clinical focus; counseling families is the primary focus of the FSCU work. Based on this focus, the FSCU team designs learning situations in which health care students and professionals can directly experience and observe conversations as well as their related pre- and post-sessions. They also create teaching units and workshops using sequences from the recorded conversations. Students develop their research skills by analyzing video-recorded FSCU material from the conversations for their MSc theses and, equally importantly, develop their clinical skills simultaneously.

## Discussion

Over the past five project years, the conceptualization and development of the FSCU have progressed. We elaborated, implemented, and advanced key work packages across the five project domains. Furthermore, the FSCU has proven to be a real-life lab for the University of Applied Sciences. Although only a few universities implemented faculty-based family nursing units in recent years, we recognize their necessity. First, the WHO’s (2015, 2016) demand for people-centered and integrated care is still being implemented, and challenges remain, including the need for new skills to effectively collaborate within networks and coordinate care (Raus et al., 2020; Sum et al., 2022). APNs can take on a leading role in such implementation programs (Schober et al., 2020), particularly as a family nursing advanced practitioner with competencies for providing leadership in working systemically (IFNA, 2017). Second, FSC is integral to people-centered and integrated care (WHO, 2015, 2016).

Integrating FSC into the development of people-centered and integrated care is important because individuals and families are often confronted with fragmented health care, as outlined in the case vignette. Integrated care counteracts the fragmentation of health care, which is associated with higher hospital use, comorbidities, and health care costs (Joo, 2023). In complex care situations, an FSC approach, in general and family nursing advanced practice in particular, should be provided throughout the illness and care trajectories to consider patient preferences, family caregiver needs, and family strengths and values (IFNA, 2017; Kokorelias et al., 2019). Thus far, families or individuals using the FSCU service have faced highly complex situations, such as having more than one severe chronic condition or experiencing health and social vulnerabilities that culminate within a single family, as exemplified in a recently published single case study (Wildhaber et al., 2025). To our knowledge, such complex family situations and how to effectively support the families in their day-to-day management have hardly been investigated.

Although FSC has been adopted in various care settings, it has primarily been implemented in pediatric and end-of-life care due to the patients’ increased dependency (Hriberšek et al., 2024). However, our case vignette reveals the needs of a family in an end-of-life situation in which the older person was still managing their life independently. This case vignette illustrates the challenges and burdens a multigenerational family faces due to a lack of attention from health care professionals regarding a health-related need. Therefore, we support Rolland’s (2025) recommendation to explore multigenerational strengths and vulnerabilities when families are confronted with illness, crisis, or loss.

Moreover, Yan et al. (2023) identified a need for interprofessional support and counseling for families before and after MAiD for a family member. However, the case vignette reveals a topic that evokes strong, ambivalent emotions for both families (Singer et al., 2023) and health care professionals, particularly nurses (Dholakia et al., 2022; Pesut et al., 2020). Experiencing a family member suffering over a prolonged period while awaiting death can burden entire families and affect bereavement, even more so if confronted with poor communication with health care professionals (Singer et al., 2023). Discussing with other professionals, as is the case within the FSCU team and in the educational activities developed in this project, can be a powerful option for nurses and other health care professionals to reflect on their own ambiguous emotions and beliefs in consideration of the disciplinary core values (Pesut et al., 2020).

This project confirmed that the demand for health-related, family-systemic therapeutic conversations in the population exceeds the current FSCU’s resources. This demand indicates a need for health care services and professionals who recognize the needs and suffering of families with health-related burdens and who support individuals and their relatives or other important persons as a unit of care (Shajani & Snell, 2023). This demand was confirmed in a Swiss Federal Office of Public Health (2020) research program. The FSCU contributes to providing services for families in gaps in the health care system and to the education and training of health care students and professionals in purposeful collaboration with families. However, the co-occurrence of health and social challenges in some families confirms the need for collaboration between health and social care professionals (Wildhaber et al., 2025). This need was also found in an umbrella review by Smith et al. (2020). Although families visiting the FSCU often already have contact with social counselors or institutions, this aspect should be considered more systematically in advancing FSC clinical practice, education, and research.

Integrating FSC into health care is important to ensure patient safety and quality of care by improving communication, coordination, and participation (Hriberšek et al., 2024). Effective communication skills must be learned (Kokorelias et al., 2019). In the FSCU real-life lab, the SHS faculty practice unit utilizes up-to-date video technology and a one-way window to enable new teaching methods for developing participatory communication skills with families. This approach aligns with the higher education need to provide learning opportunities with experts as role models, facilitating the students’ professional and personal growth, and preparing them to become multipliers in clinical practice (Jonnergård et al., 2024). FSCU lecturers, researchers, and clinicians who have specialized in FSC have expanded their teaching and research activities to include clinical practice with families experiencing real-life difficulties during the project realization. They use this expanded expertise to become role models for health care students and professionals as FSC multipliers and learn from fundamental interactions between families and health care professionals for educational and research purposes. Students and professionals deepen their knowledge and skills in FSC by observing FSC experts, actively participating in the clinical team’s pre- and post-sessions, and analyzing video-recorded real-life therapeutic conversations. We are currently examining the benefits of this teaching approach in the context of this project’s evaluation (Huber et al., 2024).

The project’s success can be attributed to the combination of project management strategies and an action learning approach. Due to the project’s high complexity, both the project management strategies and the participatory action learning approach are significantly important. The project management strategies provided a clear structure for executing the work packages across the five project domains. According to the PMBOK Guide (2021, p. 4), projects are expected to “create value for the organization and stakeholders.” Thus, stakeholder support and engagement throughout and beyond the project’s duration have been of the utmost importance. For instance, we embedded our vision for this project into the SHS mission. Building a collaborative project team is another important aspect of successful project management (PMBOK Guide, 2021). Action learning has helped to develop and sustain a collaborative project team. It has supported the project leaders and team in moving forward and bringing their vision to life by integrating the multiple perspectives of all those involved, while advocating for and engaging with the target group in need of the project: burdened families and current and future health care professionals (Zuber-Skerritt & Wood, 2019).

## Strengths and Limitations

This project has its strengths and limitations. First, many families have found support and relief from their suffering. In addition, many health care students and professionals have received education and training in FSC in addition to the traditional learning activities. Furthermore, we consider the project’s integration into the SHS strategy, support from the IN head, and careful planning to be strengths. Another strength is that we recruited experienced specialists with diverse professional and personal backgrounds for this project who networked within and outside the SHS. From the beginning, we benefited from the FSC pioneers’ many years of experience, and they provided us with professional support.

One limitation is that, despite planning from the beginning, we have not yet established the citizens’ board, the local partner’s board, or the student board due to limited time and workforce resources. These boards would have provided the FSCU with broader expertise and more comprehensive shared knowledge based on collaborative participation. Another limitation is that we realized collaboration with the other SHS institutes only partially. Collaboration with other university departments is still lacking. This limits the benefits of interprofessional collaboration between the FSCU, the SHS, and the university. Furthermore, limited staff, financial, and spatial resources restrict the number of therapeutic conversations offered to families, the number of students and professionals who can participate in the FSCU real-life lab, and the number of research projects that can be realized. Finally, we have only recently begun collaborating with local partners and other universities. This collaboration could spread and advance FSC nationally.

## Conclusion

In conclusion, the WHO’s (2015, 2016) strategy for people-centered and integrated care must be pursued. People-centered care should include families as the unit of care and complement the existing health care services with clinical counseling or therapeutic conversations for families affected by health-related challenges and burdens. Health care professionals should be educated and trained by learning from FSC experts. Research helps us to understand families better and how FSC works.

In this project, introducing family-systemic therapeutic conversations in real-life clinical work with burdened families has been central to realizing the FSCU and is unique to the Swiss context. The success of such a project depends on a well-thought-out project management plan aligned with the university’s and department’s strategies. Project management requires suitable stage planning that links the project plan’s analytical insights to actionable outcomes and thoroughly tests the counseling setting. It is also important to carefully plan for the project’s long-term sustainability. The success of the FSCU project is ultimately due to a well-functioning, motivated team that enables therapeutic conversations in a real-life teaching and learning university setting. This approach softens families’ suffering, maximizes student benefits, and allows research activities to advance further, thereby multiplying the FSCU model.
